# A Randomized Controlled Trial Investigating the Effects of a Special Extract of *Bacopa monnieri* (CDRI 08) on Hyperactivity and Inattention in Male Children and Adolescents: BACHI Study Protocol (ANZCTRN12612000827831)

**DOI:** 10.3390/nu7125507

**Published:** 2015-12-02

**Authors:** James D. Kean, Jordy Kaufman, Justine Lomas, Antionette Goh, David White, David Simpson, Andrew Scholey, Hemant Singh, Jerome Sarris, Andrea Zangara, Con Stough

**Affiliations:** 1 Centre for Human Psychopharmacology, Swinburne University of Technology, Hawthorn 3122, Australia; jkean@swin.edu.au (J.D.K.); jlomas@swin.edu.au (J.L.); agoh@swin.edu (A.G.); dwhite@swin.edu.au (D.W.); dsimpson@swin.edu.au (D.S.); ascholey@swin.edu.au (A.S.); jsarris@unimelb.edu.au (J.S.); az@naturalpowermeds.com (A.Z.); 2 Swinburne Babylab, Swinburne University of Technology, Hawthorn 3122, Australia; jkaufman@swin.edu.au; 3 Central Drug Research Institute, Lucknow 226001, India; hemant1943@gmail.com; 4 Department of Psychiatry, University of Melbourne, Parkville 3010, Australia; 5 NaturalPowerMeds Consulting SL, Barcelona 08003, Spain

**Keywords:** bacopa monnieri, CDRI 08, ADHD, inattention, hyperactivity, cognition, RCT

## Abstract

Clinical diagnoses of Attention Deficit Hyperactivity Disorder (ADHD) and the use of prescription medications for its treatment have increased in recent years. Current treatments may involve the administration of amphetamine-type substances, a treatment path many parents are apprehensive to take. Therefore, alternative pharmacological treatments are required. Few nutritional or pharmacological alternatives that reduce ADHD associated symptoms (hyperactivity and inattention) have been subjected to rigorous clinical trials. *Bacopa monnieri* is a perennial creeping herb. CDRI 08 is a special extract of *Bacopa monnieri* which has been subjected to hundreds of scientific studies and has been shown in human randomized controlled trials (RCTs) to improve memory, attention, and mood. It is hypothesised that chronic administration of CDRI 08 will improve attention, concentration and behaviour in children with high levels of hyperactivity and/or inattention. This paper reports the protocol for the first 16-week, randomized, placebo-controlled, double-blind, parallel groups trial examining the efficacy and safety of CDRI 08 in male children aged 6–14 years with high levels of inattention and hyperactivity. The primary outcome variable will be the level of hyperactivity and inattention measured by the Conners’ Parent Rating Scale (CPRS). Secondary outcome variables include cognition, mood, sleep, and EEG. Trial registration: Australia and New Zealand Clinical Trials Register (ANZCTR): ACTRN12612000827831.

## 1. Introduction

### 1.1. Background & Rationale

The first description of hyperactivity was reported by Crichton (1798) as “*mental restlessness*” [1]. However it was not until Still (1902) that a cluster of symptoms would come to be most commonly associated with a modern diagnosis of Attention Deficit Hyperactivity Disorder (ADHD) [2]. In 1937, stimulant medication was developed to treat the disease [3], officially making ADHD one of the first psychiatric disorders to be recognized, diagnosed and treated in children [4]. ADHD, Autism Spectrum Disorder (ASD), Oppositional Defiant Disorder (ODD), and Conduct Disorder (CD) are developmental disorders that may include symptoms of inattention, hyperactivity, impulsivity, and aggression beginning in childhood. Previous classifications of childhood ADHD have highlighted the perceived severity of associated symptoms with earlier diagnostic terms including Minimal Brain Damage, Minimal Cerebral Palsy, Mild Retardation, Minimal Brain Dysfunction, Hyperkinesis, Atypical Ego Development, Attention Deficit Disorder (ADD), and, finally, Attention-Deficit/Hyperactivity Disorder [5]. Recent debate regarding the over diagnosis of ADHD has shown an increase in negativity from parent groups [6], mainstream media [7], and the research community [7,8,9]. Most issues surround what is publicized as the unnecessary pharmacotherapy intervention in a vulnerable population [10]. Stimulant medication regulates neurochemical deficiencies via increasing the catecholamines dopamine (DA) and norepinephrine/noradrenaline (NE/NA), specifically within the prefrontal cortex (PFC) [11]. The PFC is largely responsible for specific cognitive functions, including executive function, attention and elements of impulsivity and hyperactivity [12]. Stimulant medications Methylphenidate (MPH) and Dextroamphetamine (DEX) exert their effect by increasing the availability of NE and DA within the PFC. However, these medications can vary in their efficacy depending on the genetic makeup of the child [13], the severity of the illness [14] and the compliance of the child and parental unit [15], and may also bring with them a number of unwanted side effects [16,17]. Such side effects can negatively impact a parent’s decision regarding best treatment options, which can lead to making uninformed alternative treatment choices [16].

Complementary, alternative and integrative medicines (CAIM) are a common treatment option for parents with children finding it difficult to focus at school, that exhibit behavioural issues, or that present with mild to moderate learning difficulties [18]. Prior studies have found that half of all parents with children diagnosed with ADHD give their child CAIM without the proper consultation with their child’s physician [19]. This highlights the necessity for randomized controlled trials to examine the potential benefits and risks of prospective complementary medicines in the treatment of ADHD symptoms and facilitate evidence based recommendations of specific CAIM in clinical and subclinical populations.

### 1.2. What is the Significance of Clinical and Sub-Clinical Symptoms of ADHD?

Clinical ADHD affects a minimum 5% of children in the Western world and remains the most common psychiatric illness among young children [20] with an estimated 50% of sufferers retaining ADHD symptoms for the rest of their lives [21,22]. Criteria used to establish a diagnosis of ADHD include the symptoms being present and persistent for a minimum of six months prior to the age of 12, are maladaptive, not consistent with the child’s developmental level and cannot be explained by other psychiatric or medical disorders [23]. A definitive underlying biological origin still eludes researchers with the principle hypothesis being a neurochemical dysfunction within the PFC. There are also parallel neuro-circuitry based theories denoted as developmental deviation [24,25] and maturational lag [26]. Using a technique known as *Neurometrics*, Quantitative EEG (QEEG) analysis revealed a deviation in the neurophysiologic functioning of ADHD children with a suggestion of hypoactivity and hyperactivity in various cortical regions [24]. Once all cortical regions are fully developed, this deviation is complete, meaning that, in at least some cases, ADHD children will have permanent neurophysiological abnormalities in certain brain regions [27,28]. Conversely, because the frontal lobes are the last cortical structures to develop, researchers have argued that the progression of brain development in ADHD children is slower when compared to normal children [29]. This “delay” can often be seen to dissipate during a second maturational peak between 17 and 21 years of age indicating that children with ADHD have “caught up” with their normally developed counterparts [29]. Both of these examples indicate neurophysiological explanations for symptoms of ADHD, and follow up studies have highlighted the capability of pharmacotherapy in reducing associated symptoms [30,31].

In the subclinical domain, children may demonstrate heightened levels of inattention and hyperactivity either at home, school, or both, but may not meet the criteria for a formal diagnosis of ADHD. Despite this, their symptoms can still cause significant issues in social and family relationships, leaving parents with relatively few options for treatment. This broader spectrum of behavioural disorders covers a vast area of developmental dysfunction that is generally ignored due to the lack of diagnostic criteria. Previous research on subclinical depression highlights the importance of recognizing the presence of symptoms in the absence of diagnosis [32] and has sparked investigations into better treatment options for children with hyperactivity and inattention than those currently available [33].

### 1.3. Bacopa Monnieri (“Brahmi”)

**Figure 1:**
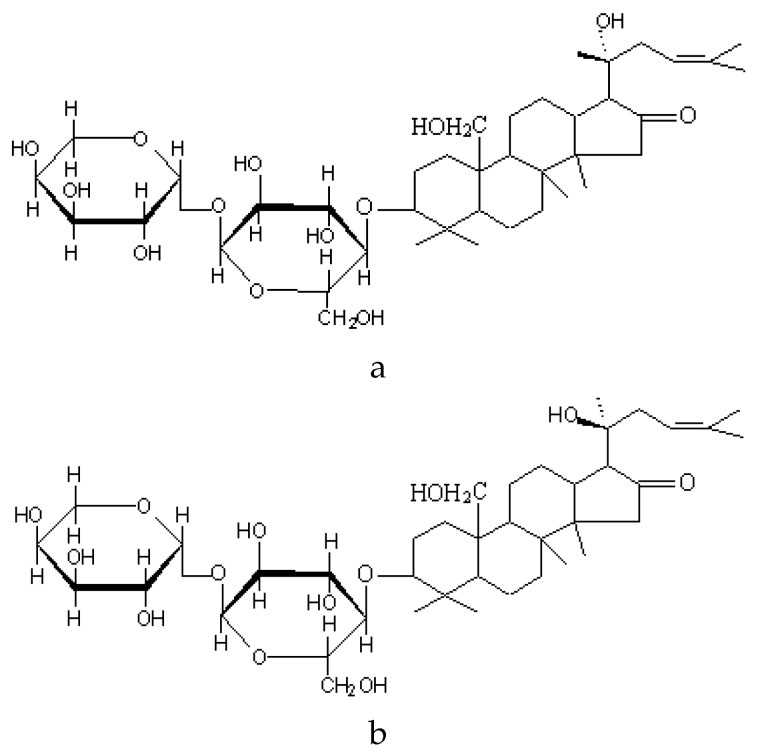
(**a**) Bacoside A (Levorotatory) [38]; (**b**) Bacoside B (Dextrorotatory) [38].

*Bacopa monnieri* (L.) Wettst. (syn. *Bacopa monnieri* Hayata & Matsum), or “*Brahmi*”, from the family Scrophulariaceae, is a perennial creeping herb that thrives in damp soils and marshes throughout the subcontinent. The name brahmi derives from the word “Brahma”, the mythical “creator” in the Hindu pantheon [34]. Bacopa has been used in the *Ayurvedic* medicinal system for approximately 3000 years and is classified as a medhyarasayana, a drug used to improve memory and intellect (medhya) [35]. *In-vivo* studies have investigated an alcoholic extract of Bacopa in albino rats showing significant improvements in the areas of learning, memory and memory retention [36,37]. The active components attributed to increased cognitive functioning in *Bacopa monnieri* are *bacosides A* and *B* (see Figure 1a,b below) [38]. Pharmacological effects can also be attributed to a number of alkaloids, saponins and sterols.

*In-vivo* studies have examined interventions with an extract containing only these bacosides, and similar significant improvements in the areas of memory and learning were found [39]. The mechanisms of action on the central nervous system include the modulation of cholinergic densities [40] and acetylcholine levels [41], β-amyloid scavenging properties [42], as well as anxiolytic processes [43,44,45,46]. The reputed effects of Bacopa have been studied in healthy adult populations with mostly positive results in terms of cognition [47,48,49,50]. Current research is examining the efficacy of Bacopa on age-related cognitive decline in the elderly and as a treatment for some types of dementia [51]. Conversely, little research has focused on the efficacy of Bacopa in younger cohorts, and even fewer in child and adolescent clinical or sub-clinical populations. Most research in younger clinical populations has emerged from Indian research groups with data derived from single extract variations and combination formula preparations [52,53,54]. Single extract studies (specifically CDRI 08) have demonstrated significant efficacy on child and adolescent ADHD symptoms [52,55,56].

### 1.4. CDRI 08

CDRI 08 is a high quality extract of *Bacopa monnieri* standardized to contain not less than 55% bacosides (based on UV spectrophotometry). The mechanisms underpinning the action of CDRI 08 have been extensively studied at the Central Drug Research Institute (CDRI-CSIR) in India. Chemical investigation of Bacopa leaves was first published in 1944 and this was followed by a chemical examination of the whole plant by researchers at CDRI in 1960, 1963 and 1967 [57]. CDRI scientists found two triterpenoid glycosides denoted *bacoside A*, which was a mixture of three compounds A2, A2’, and A3, and *bacoside B* [57]. A standard fraction containing no less than 60% bacoside A was prepared and tested in animal models examining learning, memory, and behaviour. A standardized extract of Bacopa was formerly launched by the Indian Prime Minister Narsimha Rao and made available in 1996 (then known as *Memory Plus™*) [37]. This was followed by its commercialization through Lumen Marketing (Chennai, India) in 2002 (then called *ProMind™*) [58]. In 2009, the Australian company Flordis (subsidiary company of *Soho Flordis International Pty Ltd*) launched a CDRI Bacopa extract derived from the most successful formulation ethanol extraction method, CDRI 08. The Bacopa monnieri plant is harvested twice a year by hand and is analyzed before shipment through taxonomic evaluation, a chemical analysis of the active plant ingredient through spectrophotometry, and *high performance liquid chromatography* (*HPLC*) analysis. An estimate of active compounds is quantified. The dried whole plant is extracted with ethanol to produce the CDRI08 extract, which is standardized for the active components. The product (KeenMind^®^) contains 160 mg of CDRI 08 extract, supplied in a capsule containing commonly used excipients. The herb to extract ratio for herbal products is calculated from the input of whole plant and output of extract, and can naturally vary based on several factors, such as geographic location, time of harvesting, drying process and moisture content. The CDRI 08 extract ratio is standardized to not less than 55% bacosides, providing consistency between extract batches. As an example, if 200 kg whole plant is initially used, and from this 10 kg extract is the result, the extract ratio is 20:1—this can naturally vary from 20:1 to 25:1.

A recent systematic review by Pase *et al.* (2012) has indicated that of nine clinical, double blind placebo controlled trials in humans, eight have demonstrated a positive memory, attention or cognitive enhancing effect of a Bacopa extract, with the greatest evidence for efficacy with the CDRI 08 extract in adults; some studies have also documented an anxiolytic or improved mood effect [50]. Both mood and cognitive processes are central to inattention and hyperactivity.

## 2. Experimental Section

### 2.1. Design

This is a placebo-controlled, double-blind, parallel groups (placebo *vs.* CDRI 08), randomized trial. Participants will consume 1 × 160 mg capsule of either Bacopa or placebo (if between 20 and 35 kg; to be taken with breakfast) or 2 × 160 mg capsules of either Bacopa or placebo per day (if over 35 kg; 1 to be taken with breakfast, 1 to be taken with dinner) for 14 weeks. There will be a one week placebo run-in and one week placebo run-out extending the study to a total of 16 weeks.

### 2.2. Study Aims and Hypotheses

Building upon 18 years of human research into the potential effects of the unique CDRI 08 extract [47,49,50,59], the primary aim of the current trial is to examine whether a 14 week administration of CDRI 08 improves a range of behavioural, cognitive, mood, sleep, and neurophysiological measures in males aged 6–14 years with symptoms of inattention and hyperactivity relative to placebo. The primary hypothesis is that those in the CDRI 08 treatment group will show increased levels of attention and decreased levels of hyperactivity and impulsivity when compared to those in the placebo group based on parental ratings.

### 2.3. Participants

A total of 120 healthy male participants aged between 6 and 14 years will be recruited, this number allows for a 20% dropout rate with a minimum final sample size of 100. Participants will be healthy, non-smoking males aged between 6 and 14 years, have a DSM-IV ADHD rating score above 15, be fluent or able to speak confidently in English, be accompanied by a parent or legal guardian who can provide a personally signed and dated informed consent indicating that they have been informed of all pertinent aspects of the trial, and the participant will provide a signed copy of a simplified children’s consent form. Participants will be excluded if they do not meet the above criteria and if they have a medical diagnosis other than ADHD, Oppositional Defiant Disorder or similar behavioural disorder; if they are currently taking any medication (including herbal supplements (e.g., Ginkgo) and/or stimulant medication as part of a treatment for an ADHD diagnosis). However, if they are regular users (defined as daily intake for greater than 3 months) of vitamins/fish oil supplements, participants will be asked to maintain the same habits throughout the trial. Participants will be excluded if they have a history of or have a current heart disease, high blood pressure, diabetes, a health condition that would affect food metabolism including: food allergies, kidney disease, liver disease and/or gastrointestinal diseases (e.g., irritable bowel syndrome, coeliac disease, peptic ulcers). Participation is dependent on the child and parent/guardian being able to participate in all scheduled visits, treatment plans, tests and other trial procedures according to the protocol. The child will be excluded if they do not meet the minimum cut-off on the WISC-IV-SF (<80). Finally, the child will be excluded if they are currently participating or have participated in another clinical trial during the last 2 months prior to beginning this study. Following the conclusion of the study, all participants will receive a 14 week supply of CDRI 08 to ensure participants allocated to the placebo group have the opportunity to take the extract for the same duration. The study was ethically approved by the Swinburne University Human Research Ethics Committee (SUHREC 2011/283) in collaboration with the Royal Children’s Hospital Research Ethics Committee (RCH HREC 32205 F) and all participants will provide written informed consent. The trial has been registered with the Australian and New Zealand Clinical Trials Registry (ANZCTR: 12612000827831). Laboratories are body protected and meet the requirements of the Australian standard AS:3003:1999. Biomedical equipment also conforms to the Australian standard AS:3551:1996.

### 2.4. Centre

All testing will take place within the custom built clinical trial laboratories at the Swinburne Centre for Human Psychopharmacology, Swinburne University in Victoria, Australia. 

### 2.5. Procedure

**Figure 2:**
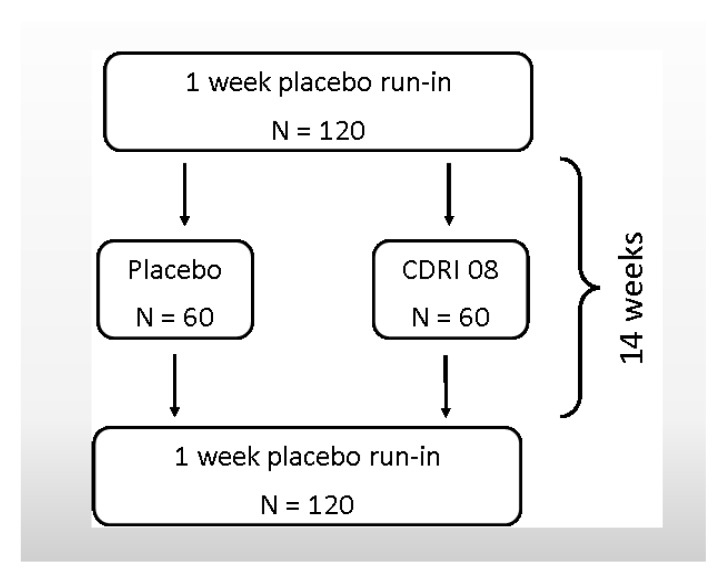
CDRI 08 protocol flow diagram.

Eligible participants will be invited to attend Swinburne University in Hawthorn for their first visit (V1; Week 0), which is a practice day where participants complete screening questionnaires and familiarise themselves with the study procedures and tests. At the end of this first visit, participants will be given a one week supply of placebo capsules to consume over the following seven days. This “placebo run-in” is in place to control for an initial placebo effect that can be observed in parental/guardian ratings of behaviour. The next visit (V2; Week 1) is a baseline session where participants complete all tests and are randomly allocated to receive one of two treatments (CDRI 08/placebo) for the next 14 weeks. Testing for V3 and V4 will follow the same outline of their baseline session, at the end of Week 15 (V4), all participants will be switched back to placebo for a period of one week with a final visit (V5) at Week 16. Each testing session will take approximately two hours. Figure 2 describes the flow of visits and Table 1 highlights the specific time points for data collection for this study.

**Table 1:** Measures and Visit Schedule.

**Behavioural, Demographic & Genetic Measures**	**V1 Screening Weeks 0**	**V2 Baseline Weeks 1**	**V3 Follow-up 1 Weeks 8**	**V4 Follow-up 2 Weeks 15**	**V5 Final Visit Weeks 16**
	Administer 1 week dose of placebo to all participants	Give randomized treatment	Continue to take randomized treatment	Give 1 week placebo run out	Return any remaining capsules
Structured interview (DSM ADHD rating)	X				
Wechsler Intelligence Scale for Children Short Form (4th Revision)	X				
Conners’ Parent Rating Scale	X	X	X	X	X
Global Clinical Impression Scale		X	X	X	
Current Health & Medical Questionnaire	X	X	X	X	X
Demographics Questionnaire	X				
OraGene Genetic Sample		X			
Cognitive & Mood Measures	V1 Practice Week 0	V2 Baseline Week 1	V3 Follow-up 1 Weeks 8	V4 Follow-up 2 Weeks 15	V5 Final Visit Weeks 16
Neurophysiology (EEG) resting states		X	X	X	
CNS Vital Signs (CNSVS)	X	X	X	X	X
Test of Variables of Attention (TOVA)	X	X	X	X	X
Hick Reaction Time Paradigm (Jensen’s Box)	X	X	X	X	X
Child Depression Inventory (CDI)	X	X	X	X	X
Paediatric Sleep Problems Survey Instrument (PSPSI)	X	X	X	X	X

### 2.6. Randomization & Blinding

All treatments are identical in appearance. Treatments are individually packaged into bottles with the participant identification number clearly labeled by a disinterested third party. The trial products are stored in a temperature controlled and monitored facility in accordance with the manufacturer’s instructions. To minimize bias, this study will employ both randomization and blinding. Placebo run-in (V1) and placebo run-out (V4) phases of the trial will be single-blinded as researchers will be aware of this component of the study design. Randomization of participants to treatment groups following placebo run-in will be determined by random allocation. All 120 participants will be assigned to treatment group A or B using a computer generated random number generator by a disinterested third party. Eligible, recruited participants will be assigned a participant number. The participant’s number will also correspond to the allocated treatment for that individual. Blinding will be achieved by enlisting a person outside of the project to code the treatments, and maintain the key to this code until data collection is completed. An emergency code break envelope will be provided to the principal investigator which will only be opened in case of emergency. If this occurs, the sponsor and the ethics committee will be informed. Participants’ parents will complete health and medical questionnaires at each visit to monitor for any adverse events. 

### 2.7. Primary Outcome

The *primary outcome* will be the level of hyperactivity and level of inattention rated by parents using the Conners’ Parent Rating Scale – 3 (CPRS) [60]. Parents will be required to complete a CPRS at each laboratory testing session. The CPRS is a behavioural assessment tool used to acquire observations of children and adolescents from the parents’ (or guardians’) point of view. It is most commonly used to elucidate problems associated with ADHD. The full-length version of the questionnaire contains 110 questions and provides comprehensive evaluations of inattention, impulsivity/hyperactivity, learning problems, executive functioning, aggression, and peer relations making the measure ideal for follow-up treatment monitoring [60,61,62].

### 2.8. Secondary Outcomes

A number of psychological, behavioural, genetic, and neurophysiological measures will be collected at each time point. These measures are described below. The testing schedule can be found in Table 1.

#### 2.8.1. Cognition

Cognitive changes will be assessed using computerized programs including CNS Vital Signs (CNSVS) [63], the Test of Variables of Attention (TOVA) [64,65], and the Hicks Reaction Time Paradigm (Jensen Box) [66]. Details of the specific cognitive outcome variables are listed in Table 2. The CNSVS is a computerized neurocognitive test battery originally developed as a brief evaluation tool to offer a better insight into patients’ complaints. It is comprised of tests that are widely used in clinical settings and are known to be reliable and valid [63]. The Test of Variables of Attention (TOVA) is a 21 min continuous performance test that assesses the different aspects of attention and their role in neurobehavioural disorders. The test is non-language based that has almost no practice effect with a sensitivity of 84% (correctly identifies true neurobehavioural disorder cases) and a specificity of around 90% (ability to correctly identify normals) [65]. The Hicks Reaction Time Paradigm (Jensen’s Box) will be administered to measure choice reaction time. The standard box has a sloping face on which 8 buttons are arrayed in a semicircle, with a “home” key in the lower center. The Jensen Box is an assessment of mental speed and declines in difficulty as the measure progresses (from 8-choice reaction time, to 4-choice, to 2-choice, to a single choice) and provides a measure of rate of information processing [63].

**Table 2:** Cognitive tasks & domains.

CNSVS	TOVA	Jensen Box
Verbal Memory Test	Errors of omission (attention)	Simple Decision Time
Visual Memory Test	Errors of commission (inhibition)	Simple Movement Time
Finger Tapping Test	Response Time	2 Choice Decision Time
Symbol Digit Coding		2 Choice Movement Time
Stroop Paradigm		4 Choice Decision Time
Shifting Attention Test		4 Choice Movement Time
Continuous Performance Test		8 Choice Decision Time
Verbal Memory Test 2		8 Choice Movement Time
Visual Memory Test 2		
Paediatric Symptom Checklist		

CNSVS = CNS Vital Signs, TOVA = Test of Variables of Attention, Jensen Box = Hicks Reaction Time Paradigm.

#### 2.8.2. Mood

The Children’s Depression Inventory 2 Self-Report Form (CDI 2:SR) [67,68,69] is a 28-item assessment that looks at emotional and functional problems including subscales of negative mood and physical symptoms, negative self-esteem, interpersonal problems and ineffectiveness.

#### 2.8.3. Sleep

Pediatric Sleep Problem Survey Instrument (PSPSI) provides a robust set of sleep problem subscales which can be used for assessment of sleep concerns in a community sample as well as provide for optimal analysis of associations with other measures of childhood daytime functioning such as neurocognition and behaviour [70].

#### 2.8.4. Global Clinical Impression Scale (GCI)

Amongst the most widely used brief assessment tools in psychiatry, the GCI is a 3-item observer-rated scale that measures illness severity (GCIS), global improvement or change (GCIC) and therapeutic response. The GCIS is rated on a 7-point scale, with the severity of illness scale using a range of responses from 1 (normal) through to 7 (amongst the most severely ill patients). GCIC scores range from 1 (very much improved) through to 7 (very much worse). Treatment response ratings should take account of both therapeutic efficacy and treatment-related adverse events and range from 0 (marked improvement and no side-effects) to 4 (unchanged or worse and side-effects outweigh the therapeutic effects). Each component of the GCI is rated separately; the instrument does not yield a global score [71].

#### 2.8.5. Neurophysiology (EEG Resting States)

Participants will undergo an electroencephalogram (EEG) by trained EEG specialists for five minutes eyes open and five minutes eyes closed to measure the resting brain oscillatory activity of the participants. Estimates of absolute and relative power in the delta, theta, alpha and beta frequency bands will be derived, as well as total power. Detailed analysis will look into the theta/beta ratio and theta/alpha ratio that have been linked to symptoms of ADHD in previous literature [72]. Change from baseline, within-group and between-group comparisons will be calculated based on resting state estimates. Brain electrical activity will be recorded from 64 sites on the scalp, including all of the international 10–20 positions and sites located midway between the 10–20 locations. All electrodes will be held in place using an electrocap. EEG will be amplified and bandpass filtered prior to digitization to 12-bit accuracy at a rate of 1000 Hz. Changes in brain wave ratios in key areas of activity, specifically within the PFC, will also be examined to test the hypothesis that CDRI 08 assists in ameliorating cortical hypoarousal that has been associated with ADHD [25,73,74,75,76,77].

#### 2.8.6. Genetic Material (OraGene Saliva Kits)

Single nucleotide polymorphisms, frequently called SNPs (pronounced “snips”), are the most common type of genetic variation among people. Each SNP represents a difference in a single DNA building block, called a nucleotide. They can act as biological markers, helping scientists locate genes that are associated with disease. When SNPs occur within a gene or in a regulatory region near a gene, they may play a more direct role in disease by affecting the gene’s function. Participants will supply a small amount of saliva in a container for closer genetic examination. Capping the container releases DNA-preserving fluid which then mixes with saliva enabling long-term storage (up to 5 years) at room temperature [78,79]. Participants are under no obligation to provide samples for genetic analysis and may choose to decline to provide a sample without consequence. In this study, we will be looking for variations within each participant’s genetic code with greater focus on SNPs associated with catecholamine and cholinergic function, as these have been linked to the symptoms of ADHD and the mechanisms of action of Bacopa, respectively [80,81]. Once collected from participants, all samples will be stored at room temperature in the medical room in the Centre for Human Psychopharmacology medical laboratory and picked up in batches by the lab performing the genetic analysis.

#### 2.8.7. Wechsler Intelligence Scale for Children Short Form (WISC-IV-SF)

According to the WISC-IV-SF Technical Manual (Wechsler, 2003b), children with ADHD may perform worse on measures of processing speed and working memory than on measures of verbal and perceptual ability [82]. Given the limited research on the WISC-IV-SF for adolescents with ADHD, the present exploration will provide data on the relationship between different aspects of IQ and impulsivity and inattention.

#### 2.8.8. Compliance, Health & Medical Assessments

Each parent will be given a treatment compliance diary and will need to mark each day that their child takes their treatment. Additionally, at the end of the study they will be asked to return all unused capsules which will be counted for compliance rate. Participants must take at least 80% of their allocated treatment. Participants’ general health and medical questionnaires will be assessed each visit to monitor for any adverse events.

### 2.9. Power Analysis

Power analysis was conducted using G*Power 3.1.2. For a repeated measures’ design with 2 groups (treatment *vs.* placebo) and 3 time points (2 weeks, 9 weeks, 15 weeks), it was determined that there would be an 80% chance of discovering a medium effect size difference (f = 0.25) between treatment groups with a total sample size of 86 participants (alpha level = 0.05). It was determined that there would be an 80% chance of discovering a medium effect size (f = 0.25) interaction between treatment group and time points with a total sample size of only 40 participants (alpha level = 0.01). With two groups of 25 participants, the study will be adequately powered (72%) to detect small changes in the primary outcome measure, the CPRS. As this study will be recruiting 120 participants, there will be sufficient power to justify any statistical significance between treatment group outcomes.

Previous research has found statistical significance when using Bacopa on cognitive processes with similar sample sizes [49]. Child and adolescent studies examining the efficacy of Bacopa have used smaller sample sizes (*n* = 40, 28, 36, 31) [52,55,56,83] and have each demonstrated statistically significant effects on cognition. Given this is the first known trial of its kind, the sample size was selected on the basis of previous effect sizes from RCTs administering Bacopa.

### 2.10. Statistical Analysis

The primary analysis will investigate the effects of Bacopa monnieri on endpoint CPRS scores of inattention and hyperactivity using repeated measures Analysis of Variance (ANOVA) methods. Other more powerful statistical techniques such as linear mixed modeling and intention to treat analysis will be considered. The ANOVA will utilize a Treatment (Placebo, Bacopa monnieri) × Time (2 weeks, 9 weeks, 15 weeks) design. Lower order interactions will be explored as appropriate utilizing one-way ANOVAs and pre-planned comparisons of treatment and time. The same 2 × 3 repeated measures ANOVA will be used to analyze all secondary outcomes including cognitive, mood, sleep, and neurophysiologic variables. Correlations and regression models may be used to examine baseline associations between variables. Results will be considered statistically significant at *p* < 0.05 corrected for multiple comparisons. Covariates such as age, gender and baseline screening measures (e.g., Wechsler intelligence) will be adjusted for in the analyses.

## 3. Conclusions

Attention Deficit Hyperactivity Disorder (ADHD) is the most common neurodevelopmental disorder among school-aged children in the Western world [4,21]. Heightened symptoms of inattention, hyperactivity, impulsivity, poor learning, executive function, aggression, and impairments in peer and home relationships have shown to increase the risk of poor childhood cognitive development [22]. Discussions regarding the over diagnosis of ADHD have become more prevalent recently [7], and there is increasing concern regarding the rapid rise in the prescription of pharmacotherapy interventions [84]. Prior studies have found that half of all parents with children diagnosed with ADHD will give their child CAIM without the proper consultation of their child’s physician [19]. This highlights the necessity for large, randomized, controlled trials such as the one described here, to examine the potential benefits of prospective complementary medicines in the treatment of ADHD symptoms in child and adolescent clinical and subclinical populations. Clinical trials such as this can facilitate evidence based recommendations of specific CAIM for symptoms of inattention and hyperactivity.

In the current study, we will administer either placebo or a novel and standardised *Bacopa monnieri* extract CDRI 08 to children and adolescents with ADHD or high levels of inattention and hyperactivity for 14 weeks. Preliminary open trials in India have indicated that CDRI 08 may be of benefit for children with behavioural problems and symptoms associated with ADHD [52,55,56,83]. Additionally, many studies have now shown that chronic administration of Bacopa improves attention and cognition in adults (children with inattention and hyperactivity show deficits in attention and cognition). The results of the study will aid our understanding of the efficacy and safety of a specific Bacopa extract (CDRI 08) in improving symptoms of inattention, hyperactivity, and impulsivity in clinical and/or subclinical populations of children and adolescents. The study results may also assist us in better understanding the mechanisms of action of this special extract of Bacopa on brain and cognitive function.
